# Effects of exogenous enzymes and dietary energy on performance and digestive physiology of broilers

**DOI:** 10.1186/2049-1891-4-14

**Published:** 2013-04-04

**Authors:** Jialing Zou, Ping Zheng, Keying Zhang, Xuemei Ding, Shiping Bai

**Affiliations:** 1 Institute of Animal Nutrition, Engineering Research Center for Animal Disease-Resistance Nutrition of China Ministry of Education, Sichuan Agricultural University, Ya'an, Sichuan, People’s Republic of China

**Keywords:** Broilers, Digestive physiology, Exogenous enzymes, Growth performance

## Abstract

The study was conducted to compare the effects of XG with AG and BM at different metabolizable energy diets on growth performance, digestive physiology and energy utilization of broilers fed with corn-SBM diet. A 2 × 4 factorial design was used with two basal diets (the positive control group, PC; negative control with ME reduction 100 kcal/kg, NC) and with or without the addition of three exogenous enzymes (0.02% BM; 0.01% AG; 0.05% XG) respectively. 1,200 one-day-old broilers were randomly allocated to 8 treatments with 10 pens of 15 broilers. There was no significant difference on BW, BWG, and FI at 0-21d, 21-42d or 0-42d for diet, enzymes or their interactions, but FI at 22-42d and 0-42d were tend to be decreased with the addition of enzymes. The F/G was significantly improved by the addition of enzymes especially in NC diet. The dietary AME and TME in PC or NC diet were significantly increased by XG or AG in NC diet. The villus length and V/C of ileum were significantly increased by the addition of BM or XG. XG improved the activities of trypsin, chymotrypsin and amylase, BM improved the activity of trypsin at 21d, and AG improved the activity of chymotrypsin at 21d. Comparing to PC diet, the addition of enzymes in PC or NC diet decreased feed cost per kg body weight gain especially in NC diet (except AG in PC diet) with the highest profits for XG in NC diet. In conclusion, supplementation of 0.02% BM or 0.01% AG or 0.05% XG could improve feed conversion of broilers in corn-soybean meal diet by improving energy utilization and digestive physiology, and also supplementation of 0.05% XG had a preferable efficacy in low energy diet.

## Introduction

The identification and alleviation of factors that inhibit nutrient utilization are necessary for poultry production. The nonstarch polysaccharides (NSP) in ingredients such as soybean meal and sesame meal are the main factors which reduce nutrient bioavailability [1]. The NSPs include various fiber types such as lignin, arabinoxylan, *β*-glucans, galactose and mannose in poultry feedstuffs [2]. Corn-SBM diet mainly used in poultry was assumed to cause no digestive problem in poultry, so the exogenous enzymes were not required. But researchers have proved that corn-SBM diet contains numerous antinutritional factors, such as *β-*glucans, *β-*mannose, protease inhibitors and lectins, and it has been proved that the addition of exogenous enzymes in corn and soybean meal is justified and feasible [3].

In our study, BM is a commercial *β*-mannanase product, AG is a commercial galactosidase product and XG is a new commercial enzyme product containing endo-xylanases and *β*-Glucanase. Studies have demonstrated that *β*-mannanase in corn-SBM diet improved growth performance and energy utilization in broilers [4]. AG is used in poultry diet containing soybean meal with improved growth performance of broilers and nutrient availability of soybean meal [5,6]. Owusu-Asiedu reported that the addition of mixed xylanase and *β*-Glucanase improved performance in weaned pigs [7]. And Cowieson reported that Xylanase and *β-*Glucanase improved feed conversion ratio and ileal nutrient digestibility in broilers [8].

So far, little research has been conducted to investigate the effect of XG on growth performance of broilers. A study was conducted to compare the effects of XG, AG and BM at two metabolizable energy diets on growth performance, digestive physiology and energy utilization of broilers fed with corn-SBM diet.

## Materials and methods

### Experimental design and broilers

All experimental procedures were approved by the Animal Care and Use Committee of Sichuan Agricultural University. The feeding experiment was conducted in the cage pen house of the Animal Nutrition Research Centre in Sichuan Agricultural University.

A 2 × 4 factorial treatment arrangement was used in this study with two basal diets (the positive control group with normal ME for broilers, PC; negative control with ME reduction 100 kcal/kg, NC) and with or without the addition of three different exogenous enzymes (BM, *β-*mannanase 140 × 10^6^ U/kg; AG, *α-*galactosidase 750,000 U/kg; XG, xylanase 3,000 U/kg and *β-*Glucanase 400 U/kg) respectively. Dietary treatments were shown in Table 1. The three exogenous enzymes were provided by Nutreco Nederland B.V.

**Table 1:** Experimental design and treatments

**Treatments**	**Diets**
1	NC, negative control with ME reduction 100 kcal/kg
2	NC + 0.02% BM, *β*-mannanase 140 × 10^6^ U/kg
3	NC + 0.01% AG, *α-*galactosidase 750,000 U/kg
4	NC + 0.05% XG, xylanase 3,000 U/kg and *β-*Glucanase 400 U/kg
5	PC, the positive control group with normal ME for broilers
6	PC + 0.02% BM, *β*-mannanase 140 × 10^6^ U/kg
7	PC + 0.01% AG, *α-*galactosidase 750,000 U/kg
8	PC + 0.05% XG, *α-*galactosidase 750,000 U/kg

A total of 1,200 one-day-old male Cobb broiler chicks were randomly distributed by body weight to the 8 treatments with 10 replicate pens of 15 broilers. Room temperature was kept at 33°C −35°C during the first week and gradually decreased to 24°C by the end of the third week. The chicks were given free access to feed and water with 24 h light.

The PC basal diet was corn and SBM type and formulated to meet the nutrient recommendation according to Feeding Standard of Chicken of the People’s Republic of China (NY/T 33–2004), and the compositions were showed in Table 2. The doses of the three exogenous enzymes followed the enzyme provider’s recommendation. The NC diet dietary energy was reduced by decreasing rapeseed oil level. All diets were steam pelleted with a conditioning temperature below 75°C with pellet sizes of 1.3 mm and 2.3 mm respectively for starter and grower.

**Table 2:** Composition and nutrients levels of basal diets

**Items**	**PC**		**NC**	
	**Starter**	**Grower**	**Starter**	**Grower**
	**0- 21 d**	**22-42d**	**0- 21 d**	**22-42d**
**Ingredients, %**				
Corn	52.50	60.10	52.50	60.10
Soybean meal	39.10	33.20	39.10	33.20
Rapeseed oil	3.03	2.98	1.95	1.90
CaCO_3_	0.80	0.66	0.80	0.66
CaHPO_4_	1.80	1.60	1.80	1.60
DL- Methionine	0.19	0.13	0.19	0.13
Choline chloride	0.15	0.10	0.15	0.10
NaCl	0.40	0.40	0.40	0.40
Vitamin premix^1^	0.03	0.03	0.03	0.03
Mineral premix^2^	0.50	0.30	0.50	0.30
Betonite^3^	1.50	0.50	2.58	1.58
Total	100.0	100.0	100.0	100.0
**Calculated nutrient content**				
ME, kcal/kg	2,903.85	3,001.84	2,803.47	2,903.85
Crude protein, %	21.01	19.08	21.01	19.08
Met, %	0.49	0.41	0.49	0.41
Lys, %	1.14	1.01	1.14	1.01
Met + Cys, %	0.81	0.71	0.81	0.71
Thr, %	0.80	0.73	0.80	0.73
Calcium, %	0.97	0.80	1.14	1.01
Nonphytate, % P	0.44	0.40	0.44	0.40

^1^Provided per kg of diet: vitamin A, 4 × 10^7^ IU; vitamin D_3_, 1 × 10^7^ IU; vitamin E, 25,000 IU; vitamin K_3_, 5,000 mg; thiamin, 2,000 mg; riboflavin,16,000 mg; vitamin B_6_, 6,000 mg; vitamin B_12_, 30 mg; folic acid, 500 mg; niacin, 35,000 mg; Ca-D-panthothenate, 25,000 mg.

^2^Provided per kg of diet: Fe(FeSO_4_.H_2_O),100 mg; Cu(CuSO_4_.5H_2_O);8 mg; Mn(MnSO_4_.H_2_O),120 mg; Zn (ZnSO_4_.H_2_O),100 mg; Se (Na_2_SeO_3_), 0.3 mg; I (KI), 0.7 mg.

^3^The part of betonite in the basal diet was replaced by BM, AG, or XG for different dietary treatments.

### Observations

#### Growth performance

The broilers were weighed by pens at 0, 21, and 42 d of age, feed consumption for each pen was recorded by starter (0-21d) or grower (22-42d) phase. The average body weight (BW), body weight gain (BWG), feed intake (FI) and feed to gain ratio (F/G) were recorded at starter, grower and the whole phases (0-42d).

#### AME and TME measurements

At the end of 42d, 72 healthy broilers (average body weight 2.8 kg) were selected from treatment 1 and divided into 9 groups. The broilers were fed individually in metabolism cages with 24-h light and surgically fitted with modified plastic retainer lids for the collection of feces. After a 48-h fasting, the broilers of 8 groups were force-fed by 50 g respective diets. Total excreta were collected for the following 48 h. The other group broilers were continue to fast for 48 h to collect the excreta as the endogenous nutrient loss. During the collection period, broilers were given free access to water. Excreta were weighed and frozen at −20°C until analysis. The samples of feed and excreta were analyzed for dry matter (DM) and gross energy (GE). The gross energy was measures with bomb calorimeter. The apparent and true metabolizable energy values (AME and TME) of the diets were calculated by the method described by Sibbald [9].

$$\begin{array}{l} \mathrm{AME}=\left(\mathrm{EI}-\mathrm{EO}\right)/\mathrm{FI}\\ \mathrm{TME}=\mathrm{AME}+\left(\mathrm{EEL}/\mathrm{FI}\right) \end{array}$$

where EI is the gross energy intake. EO is the gross energy in excreta. EEL is the endogenous energy loss.

### Digestive physiology

At 21 and 42 day of age, one broiler with an average weight per replicate was selected and killed by cervical dislocation. The anterior intestine (duodenum, the U-shaped bending from gizzard to jejunum) and ileum (from Meckel’s diverticulum to the cecal junction) of each broiler were collected. Then 2.0 cm-long samples from duodenum and ileum were taken to measure the villus length and crypt depth, and the villus length to crypt depth ratio (V/C) was calculated by the method of Lei [10]. Villus length and crypt depth were measured using software for image analysis (Image-Pro Plus 6.0).

One broiler per replicate was chosen and fed for 2 h. After 4 h feeding, the intestinal contents of the duodenum plus jejunum were collected and stored at −20°C for analysis of trypsin, chymotrypsin, lipase, and amylase activities according to the method of detection kit instructions from Nanjing Jiancheng Bioengineering Institute [10].

### Statistical analyses

Data were analyzed by multivariate ANOVA of SPSS 16.0 for Windows (SPSS Inc., Chicago, IL). The model included diet (PC and NC), different enzymes as the main effects. Variable means for treatments showing significant differences in the ANOVA were separated by Duncan test. In all analyses, significance was declared at *P* ≤ 0.05.

## Results

### Growth performance

Results of growth performance were presented in Tables 3 and 4. There was no significant difference on BW, BWG, and FI at 0-21d, 22-42d or 0-42d for diet, enzymes or their interaction, but the FI at 22-42d and 0-42d were tended to be decreased with the addition of enzymes. The F/G was significantly influenced by the addition of enzymes, diet (except at 0-21d), or their interaction (except at 0-21d) (*P* < 0.05). Compared to PC diet, the NC diet significantly increased the F/G at 22-42d and 0-42d (*P* < 0.05). The addition of BM (except at 22-42d), or AG, or XG significantly decreased the F/G at 0-21d, 22-42d and 0-42d (*P* < 0.05). Compared to the NC diet, addition of BM, AG or XG in NC diet significantly improved F/G by 6.1%, 5.4%, and 6.7% respectively (*P* <0.05) at 0-21d and by 6.3%, 8.1% and 8.5% respectively (*P* <0.05) at 22-42d. Compared to the PC diet, addition of BM in PC diet significantly improved F/G by 4.9% at 0-21d (*P* <0.05).

**Table 3:** Effects of exogenous enzymes and diet on the BW and BWG of broilers

**Diet**	**Enzyme**	**BW, g**			**BWG, g**		
		**0 d**	**21 d**	**42 d**	**0-21 d**	**22-42 d**	**0-42 d**
NC	no	42.4	816.5	2,590.2	774.1	1,773.0	2,547.0
	BM	42.4	830.5	2,568.7	788.1	1,736.0	2,524.0
	AG	42.4	845.7	2,662.0	803.3	1,814.0	2,618.0
	XG	42.5	848.1	2,687.2	805.5	1,837.0	2,642.0
PC	no	42.5	808.4	2,702.5	765.9	1,893.0	2,659.0
	BM	42.4	849.2	2,723.8	806.7	1,869.0	2,676.0
	AG	42.5	810.1	2,622.6	767.6	1,811.0	2,579.0
	XG	42.5	816.1	2,677.5	773.6	1,856.0	2,630.0
**Main effects**							
Enzyme	no	42.5	812.5	2,646.4	770.0	1,833.0	2,603.0
	BM	42.4	839.8	2,646.3	797.4	1,803.0	2,600.0
	AG	42.4	827.9	2,642.3	785.5	1,813.0	2,598.0
	XG	42.5	832.1	2,682.4	789.6	1,846.0	2,636.0
Diet	NC	42.4	835.2	2,627.0	792.8	1,790.0	2,583.0
	PC	42.5	820.9	2,681.6	778.5	1,857.0	2,636.0
SEM		0.02	4.97	21.32	4.98	21.23	21.35
***P*-Value**							
Enzyme		0.448	0.268	0.900	0.267	0.886	0.914
Diet		0.546	0.156	0.205	0.155	0.118	0.219
Enzyme × diet		0.620	0.198	0.313	0.199	0.578	0.322

In the same row, values with no letter or the same letter superscripts mean no significant difference (*P* > 0.05), while with different small letter superscripts mean significant difference (*P* < 0.05), and with different capital letter superscripts mean significant difference (*P* < 0.01). The same as below.

**Table 4:** Effects of exogenous enzymes and diet on the FI and F/G of broilers

**Diet**	**Enzyme**	**FI, g**			**F/G**		
		**0-21d**	**22-42d**	**0-42d**	**0-21d**	**22-42d**	**0-42d**
NC	no	1,258.9^b^	3,975.0 ^b^	5,431.0^b^	1.632^c^	2.239 ^c^	2.013 ^c^
	BM	1,206.0 ^a^	3,596.0 ^a^	4,750.0 ^a^	1.532^ab^	2.081 ^b^	1.886 ^b^
	AG	1,238.6^ab^	3,651.0^ab^	4,838.0^ab^	1.543^ab^	2.014^ab^	1.849^ab^
	XG	1,223.8^ab^	3,685.0^ab^	4,858.0^ab^	1.520 ^a^	2.016^ab^	1.842^ab^
PC	no	1,220.3^ab^	3,756.0^ab^	4,926.0^ab^	1.597^bc^	1.990^ab^	1.856^ab^
	BM	1,221.9^ab^	3,709.0^ab^	4,875.0^ab^	1.518 ^a^	1.990^ab^	1.821^ab^
	AG	1,218.8^ab^	3,625.0^ab^	4,790.0 ^a^	1.590^abc^	2.013^ab^	1.862^ab^
	XG	1,223.5^ab^	3,531.0 ^a^	4,717.0 ^a^	1.586^abc^	1.904^a^	1.796^a^
**Main effects**							
Enzyme	no	1,239.6	3,866.0 ^b^	5,028.0 ^b^	1.615 ^b^	2.115^b^	1.934^B^
	BM	1,213.9	3,652.0^ab^	4,813.0^ab^	1.525^a^	2.035^ab^	1.853^A^
	AG	1,228.7	3,638.0^ab^	4,814.0^ab^	1.566^a^	2.014^a^	1.856^A^
	XG	1,223.7	3,608.0 ^a^	4,788.0 ^a^	1.553 ^a^	1.960 ^a^	1.819^A^
Diet	NC	1,231.8	3,727.0	4,894.0	1.557	2.088^A^	1.897^A^
	PC	1,221.1	3,655.0	4,827.0	1.573	1.975^B^	1.833^B^
SEM		5.14	38.84	37.59	0.008	0.014	0.009
***P*-Value**							
Enzyme		0.365	0.084	0.091	0.002	0.003	<0.001
Diet		0.302	0.357	0.372	0.331	<0.001	0.001
Enzyme × diet		0.275	0.451	0.440	0.097	0.027	0.013

## TME results

Result for AME, TME, and dry matter output were presented in Table 5. The dietary AME and TME were influenced significantly by the diet or the addition of enzymes (*P* <0.05). NC diet had significantly lower AME (−8.1%) or TME (−6.9%) than PC diet (*P* <0.05). The addition of XG significantly increased the AME (+3.9% in NC diet, +14.6% in PC diet, *P* <0.05), and TME (+3.3% in NC diet, +12.5% in PC diet, *P* <0.05), while the addition of AG significantly increased AME and TME in NC diet but significantly decreased the AME and TME in PC diet (*P* <0.05). The addition of XG in PC diet significantly decreased the dry matter output (*P* <0.05).

**Table 5:** Effects of exogenous enzymes and diet on the energy utilization of diet (air dry basis)

**Diet**	**Enzyme**	**AME, kcal/kg**	**TME, kcal/kg**	**Dry matter output, g**
NC	no	2,435.2^a^	2,882.7 ^a^	19.02 ^a^
	BM	2,394.2 ^a^	2,841.8^a^	20.34 ^a^
	AG	2,516.5 ^a^	2,964.1^a^	18.97 ^a^
	XG	2,532.0^a^	2,979.6 ^a^	18.23 ^a^
PC	no	2,599.7 ^a^	3,047.1 ^a^	18.67 ^a^
	BM	2,725.7 ^ab^	3,173.3 ^ab^	18.04 ^a^
	AG	2,441.3 ^a^	2,888.9^a^	21.51 ^a^
	XG	2,979.3 ^b^	3,426.8 ^b^	14.24 ^b^
**Main effects**				
Enzyme	no	2,517.4 ^a^	2,964.9 ^a^	18.84 ^ab^
	BM	2,559.9 ^ab^	3,007.5 ^ab^	19.19 ^a^
	AG	2,478.9 ^a^	2,926.5 ^a^	20.24^a^
	XG	2,755.7 ^b^	3,203.2 ^b^	16.23 ^b^
Diet	NC	2,469.5^a^	2,917.1 ^a^	19.14
	PC	2,686.5 ^b^	3,134.0 ^b^	18.12
SEM		103.0	103.0	1.304
***P*-Value**				
Enzyme		0.045	0.045	0.024
Diet		0.004	0.004	0.272
Enzyme × diet		0.075	0.075	0.085

### Intestine morphology

Results for duodenum morphology were shown in Table 6. The crypt depth was significantly decreased by the addition of enzymes in the PC diet (−7.9%) at 21d, and decreased with the addition of AG in the PC diet at 21 d (−16.6%) (*P* <0.05). The V/C was significantly increased by PC diet (+9.7%) at 21 d, and the addition of AG increased V/C (+19.3%) (*P* <0.05) at 21d.

**Table 6:** Effects of exogenous enzymes and diet on duodenum morphology in broilers

**Diet**	**Enzyme**	**21 d**			**42 d**		
		**Villus length, μm**	**Crypt depth, μm**	**V/C**	**Villus length, μm**	**Crypt depth, μm**	**V/C**
NC	no	1,547.3^ab^	151.0 ^c^	10.37 ^a^	1,509.3^a^	152.3	9.99 ^a^
	BM	1,618.2^ab^	150.8 ^c^	10.81^ab^	1,632.4^ab^	160.6	10.31^ab^
	AG	1,373.8^a^	122.7^a^	11.14^ab^	1,609.5^ab^	144.9	11.15 ^ab^
	XG	1,582.8^ab^	149.0^bc^	10.74^ab^	1,785.1^b^	162.1	11.32 ^ab^
PC	no	1,482.6^ab^	143.2^bc^	10.40 ^a^	1,671.0^ab^	162.8	10.50 ^ab^
	BM	1,490.9^ab^	135.9^abc^	11.03^ab^	1,666.6 ^ab^	159.6	10.51^ab^
	AG	1,654.5^b^	122.5^a^	13.66^c^	1,781.5 ^b^	147.9	12.31^b^
	XG	1,633.9^ab^	130.0^ab^	12.60 ^bc^	1,499.7 ^a^	143.9	10.55 ^ab^
**Main effects**							
Enzyme	no	1,515.0	147.1^b^	10.38 ^a^	1,590.1	157.6	10.25
	BM	1,554.5	143.3^b^	10.92 ^a^	1,649.5	160.1	10.41
	AG	1,514.1	122.6^a^	12.39^b^	1,695.5	146.4	11.73
	XG	1,608.4	139.5^b^	11.67 ^ab^	1,642.4	152.9	10.93
Diet	NC	1,530.6	143.4 ^b^	10.76^a^	1,634.1	155.0	10.69
	PC	1,565.5	132.9^a^	11.92^b^	1,654.7	153.5	10.97
SEM		82.42	6.33	0.677	61.41	9.37	0.683
***P*-Value**							
Enzyme		0.631	0.002	0.028	0.408	0.489	0.145
Diet		0.553	0.024	0.021	0.637	0.830	0.571
Enzyme × diet		0.084	0.475	0.197	0.002	0.481	0.562

The data of effect of the dietary treatments on ileum morphology were shown in Table 7. The villus length, crypt depth (except at 21d) and V/C were significantly influenced by the addition of enzymes, diets (except the villus length at 42d) or their interactions (*P* <0.05). At 21d, the villus length was significantly increased by 53.7% and 40.4% with the addition of BM and XG in PC diet and the V/C was significantly increased by 72.3% and 51.8% with the addition of BM and XG. NC diet significantly decreased the villus length by 15.5% at 21d, but increased the V/C by 22.4% at 42d (*P* <0.05). The crypt depth was significantly increased by the addition of enzymes in PC diet and by PC diet at 42d. The addition of BM and XG significantly decreased the V/C in PC diet, while significantly increased by AG at 42d (*P* <0.05).

**Table 7:** Effects of exogenous enzymes and diet on ileum morphology in broilers

**Diet**	**Enzyme**	**21 d**			**42 d**		
		**Villus length, μm**	**Crypt depth, μm**	**V/C**	**Villus length, μm**	**Crypt depth, μm**	**V/C**
NC	no	949.5^a^	124.8^a^	7.69^b^	953.0 ^a^	124.4 ^ab^	7.66 ^ab^
	BM	863.5 ^a^	139.6^ab^	6.31 ^ab^	1,140.1^a^	136.2^b^	8.34 ^b^
	AG	847.5 ^a^	151.5^b^	5.70 ^a^	1,498.1^b^	142.0 ^bc^	10.56 ^c^
	XG	965.7 ^a^	139.8^ab^	6.95 ^ab^	1,043.4^a^	138.5 ^bc^	7.50 ^ab^
PC	no	857.6 ^a^	142.7^ab^	6.14 ^a^	1,156.8^a^	101.6^a^	11.62 ^c^
	BM	1,318.4^b^	124.7^a^	10.58^c^	1,054.1^a^	149.3 ^bc^	7.09 ^ab^
	AG	913.6 ^a^	133.3^ab^	6.93^ab^	1,094.3^a^	165.0^c^	6.63 ^ab^
	XG	1,204.3 ^b^	128.7 ^ab^	9.32^c^	1,007.2^a^	192.7^d^	5.43^a^
**Main effects**							
Enzyme	no	903.6^A^	133.7	6.91^A^	1,054.9 ^a^	113.0^A^	9.64 ^C^
	BM	1,091.0^B^	132.1	8.45^B^	1,097.1 ^a^	142.8^B^	7.72^AB^
	AG	880.5^A^	142.4	6.32^A^	1,296.2^b^	153.4 ^BC^	8.59^BC^
	XG	1,084.8^B^	134.2	8.14^B^	1,025.3 ^a^	165.6 ^C^	6.47 ^A^
Diet	NC	906.5 ^A^	138.9	6.66 ^A^	1,158.7 ^b^	135.3^a^	8.52^B^
	PC	1,073.5 ^B^	132.4	8.24^B^	1,078.1 ^a^	152.2^b^	7.69 ^a^
SEM		56.16	7.49	0.463	98.37	8.61	0.722
***P*-Value**							
Enzyme		<0.001	0.523	<0.001	0.038	<0.001	0.001
Diet		<0.001	0.223	<0.001	0.254	0.008	0.116
Enzyme × diet		<0.001	0.078	<0.001	0.032	0.001	<0.001

### Digestive enzyme activities

The data of effect of the dietary treatments on digestive enzyme activities in small intestine were shown in Table 8. At 42d, the trypsin activity was significantly increased by AG or XG in NC diet, and decreased by BM in NC and PC diet (*P* <0.05). The addition of XG in PC diet significantly increased the trypsin activity at 21d (*P* <0.05). The chymotrypsin activity was significantly decreased by NC diet, and increased by XG in PC diet (*P* <0.05). And addition with AG in NC diet increased the chymotrypsin activity at 42d (*P* <0.05). At 42d, the lipase activity was significantly increased by NC diet (*P* <0.05). The addition of enzymes in PC diet tended to increase lipase activity at 21d, significantly for XG in PC diet (*P* <0.05). At 42d, the amylase activity was significantly decreased by PC diet and increased by XG in NC diet (*P* <0.05). The amylase activity at 21d was significantly increased by XG (*P* <0.05).

**Table 8:** Effects of exogenous enzymes and diet on digestive enzyme activities in small intestinum of broilers,U/mg

**Diet**	**Enzyme**	**Trypsin**		**Chymotrypsin**		**Lipase**		**Amylase**	
		**21 d**	**42 d**	**21 d**	**42 d**	**21 d**	**42 d**	**21 d**	**42 d**
NC	no	3,425.3^abc^	3,243.1^abc^	0.523 ^ab^	0.548 ^a^	427.2 ^ab^	467.3^abc^	58.15	143.4^bc^
	BM	3,465.4 ^abc^	2,501.0 ^a^	0.359 ^a^	0.659 ^ab^	362.8 ^ab^	355.7 ^ab^	59.77	119.5 ^abc^
	AG	4,837.5^bc^	4,139.0^cd^	0.905 ^b^	1.080 ^c^	473.2^b^	596.5^c^	63.18	145.1^bc^
	XG	4,264.8 ^abc^	5,978.4^e^	0.805 ^b^	0.898 ^bc^	394.7^ab^	463.9 ^abc^	75.20	192.5^d^
PC	no	2,848.4 ^a^	4,655.0^d^	0.754 ^ab^	1.064 ^c^	291.5 ^a^	499.8^bc^	59.63	160.9^cd^
	BM	4,290.9^abc^	2,760.3^ab^	0.690 ^ab^	0.726 ^ab^	426.2 ^ab^	385.1^ab^	69.53	95.06^a^
	AG	3,050.6 ^ab^	3,571.2^bc^	0.726 ^ab^	0.653 ^ab^	351.0^ab^	316.5^a^	62.67	104.5 ^ab^
	XG	5,289.4^c^	3,917.3^cd^	1.473 ^c^	1.483 ^d^	474.1^b^	361.5^ab^	78.56	116.4 ^ab^
**Main effects**									
Enzyme	no	3,136.8 ^a^	3,949.0^B^	0.638 ^AB^	0.806^A^	359.3	483.6^b^	58.90^a^	152.1 ^bc^
	BM	3,878.2 ^ab^	2,630.7 ^A^	0.525^A^	0.692^A^	394.5	370.4 ^a^	64.65 ^ab^	107.3 ^a^
	AG	3,944.0 ^ab^	3,855.1^B^	0.815^B^	0.867 ^A^	412.1	456.5 ^ab^	62.93 ^a^	124.8 ^ab^
	XG	4,777.1^b^	4,947.9^C^	1.139^C^	1.191 ^B^	434.4	412.7 ^ab^	76.88 ^b^	154.5 ^c^
Diet	NC	3,998.2	3,965.4	0.648^a^	0.796 ^a^	414.5	470.8 ^b^	64.08	150.1^b^
	PC	3,869.8	3,725.9	0.911 ^b^	0.981 ^b^	385.7	390.7 ^a^	67.60	119.2^a^
SEM		582.2	333.8	0.130	0.104	47.5	48.6	6.18	13.8
***P*-Value**									
Enzyme		0.062	<0.001	<0.001	<0.001	0.455	0.116	0.036	0.003
Diet		0.757	0.316	0.007	0.016	0.396	0.025	0.425	0.003
Enzyme × diet		0.069	<0.001	0.022	<0.001	0.043	0.007	0.854	0.014

### Economic profit

Results for economic profit were presented in Table 9. Comparing with PC group (as 100%), the addition of BM, AG and XG in NC or PC diet decreased the feed cost per kg BWG by 6.1%, 6.5%, 6.8% or 3.1%, 2.7%, 7.5%, while increased the profits (except AG in PC diets) by 0.4%, 7.8%, 9.8% or 3.8%, 3.7%, 5.9%, with the highest profits for XG especially in NC diet.

**Table 9:** Economic efficiency of the broilers (1,000 broilers)

**Diet**	**Enzyme**	**0—42 d**					**broilers**			**profits**
		**FI, kg**	**F/G**	**Feed cost**^**1**^**U.S.dollar**	**Feed cost/BWG U.S. dollar/kg**	**Compered with PC,%**	**BWG, kg**	**Income**^**2**^**U.S. dollar**	**Profits**^**3**^**U.S. dollar**	**Compared with PC,%**
NC	no	5,431.0	2.01	2,504.0	0.50	108.7	2,547.0	3,540.9	1,036.9	85.9
	BM	4,750.0	1.89	2,297.3	0.43	93.7	2,524.0	3,508.9	1,211.6	100.4
	AG	4,838.0	1.85	2,339.1	0.43	93.4	2,618.0	3,639.6	1,300.5	107.8
	XG	4,858.0	1.84	2,347.8	0.43	93.3	2,642.0	3,672.9	1,325.2	109.8
PC	no	4,926.0	1.86	2,490.1	0.46	100.0	2,659.0	3,696.6	1,206.4	100.0
	BM	4,875.0	1.82	2,467.4	0.45	97.0	2,676.0	3,720.2	1,252.8	103.8
	AG	4,790.0	1.86	2,423.7	0.45	97.3	2,579.0	3,585.3	1,161.6	96.3
	XG	4,717.0	1.80	2,379.0	0.43	92.5	2,630.0	3,656.2	1,277.2	105.9

^1^Cost of enzymes was not included.

^2^ Price of broilers was 1.39U.S. dollars/kg in the market.

^3^Profits = income- cost of feed consumption.

## Discussion

Studies have well documented that the addition of exogenous enzymes in corn-SBM diet improved broilers performance and nutrient utilization [11,12]. Daskiran demonstrated that the addition of 0.05%, 0.10% and 0.15% Hemicell, whose active ingredient was *β*-mannanase, in corn-SBM diet improved F/G by 2.8% and 5.7% at 0-2wk [13]. Zou reported that the supplementation with 0.025%, 0.05% and 0.075% Hemicell in a corn-SBM diet improved (*P* <0.05) weight gain respectively by 3.52%, 5.06% and 5.39% at 22-42d and by 2.86%, 4.64% and 3.18% at 0-42d, F/G for the 0.025% and 0.05% groups was significantly better than the control group at 22-42d (3.72% and 4.96%) and 0-42d (2.14% and 5.07%)[14]. Kidd reported that the addition of 112 g/t enzyme preparation, the active ingredient was *α*-galactosidase, in corn-SBM diet decreased F/G by 8.4% at 0–7 wk [15], while the addition of 115 g/t decreased F/G by 2.2% at 0–7 wk [16]. In present study, the addition of BM or AG improved F/G at 0-21d (by 6.1% and 5.4%), 22-42d (by 7.1% and 10.0%) and 0-42d (by 6.3% and 8.1%) in NC diet but not in PC diet (except for BM at 0-21d). It was not consistent with the previous study in BW or BWG.

Additionally, F/G of broilers fed NC diet with the addition of enzymes was similar to the PC diet with no significant difference. This was consistent with the study of Wu [17], who demonstrated that feed conversion of hens fed the low-energy diet based on corn-SBM diet supplemented with *β-*mannanase was similar to that of hens fed the high-energy diet. In our study, the effect of XG on F/G was lower than that of BM and AG, especially in NC diet or at 22-42d, which was related to the different composition of enzymes. It has been proved that the effect of xylanase and *β*-glucanase (the main ingredient of XG) in corn-SBM diet was additive [18]. Corn-SBM diet contains high level of starch (in corn) and nonstarch polysaccharides (in soybean meal), which are potentially antagonistic to nutrient utilization and negatively affect the growth performance. Therefore, supplementing in corn-SBM diet with xylanase and *β*-glucanase can hydrolyze the ploysaccharides and unlock the encapsulated starch molecules, and potentially improve the utilization of corn-SBM diet, while simultaneously improving the digestibility of nutrients, resulting in the conservation of endogenous utilizable nutrients and energy that cannot be otherwise used for protein accretion of broilers. And researchers have demonstrated that the broiler diets based on corn-SBM supplemented with xylanase and *β*-glucanase was able to compensate for some reduction in dietary ME content without compromising feed conversions [19-21].

Similar to F/G, the addition of XG in NC and PC diet significantly (*P* <0.05) increased the dietary AME, TME, and decreased dry matter output. Our study demonstrated that XG improved the energy utilization, and resulted in more energy available for broilers’ growth. The results were also similar to other studies, which showed that the addition of xylanase and *β-*glucanase improved nutrient digestibility and growth performance in ducks and chickens fed corn-SBM diet [22,23]. The addition of AG in NC diet significantly increased AME, TME, and improved F/G, but AME, TME were decreased in PC diet. AME and TME determinations were made of soybeans varying in oligosaccharide content due to ethanol extraction or from genetic selection generally, indicating that the chicks in PC group added with AG do not use these fractions well. The addition of BM decreased F/G, but showed no influence on AME and TME, which indicated that the growth performance of broilers was affected not only by nutrient utilization, but also by the immunization or health of digestive tract [24].

Mehri added 0.09% *β*-mannase in corn-SBM diet and found that *β*-mannase improved villus length, V/C, and broilers growth performance [25]. The results of present study showed that the addition of XG improved villus length, V/C (at 21d) and crypt depth (at 42d) of ileum, while BM improved villus length and V/C of ileum at 21 d, and AG improved V/C of duodenum at 21d, villus length, crypt depth and V/C of ileum at 42d. This finding was consistent with the previous studies [26]. These results provided the evidence for the improvement of diet energy utilization and F/G.

The other effect of exogenous enzymes on broiler performance may be explained by increasing the output of pancreatic juice and endogenous digestive enzyme activities. The NSP, such as *β*-glucanase and cellulase in corn-SBM diet increased the intestinal viscosity and negatively influences the digestion and absorption of nutrients. Wang added NSP degrading enzymes (including xylanase, *β*-glucanase and cellulase) in rough rice-based pig diets and showed that the activities of protease, trypsin and amylase in duodenal content were significantly increased, and the average daily gain and F/G were (*P* <0.05) significantly improved [27]. Engberg reported that with the addition of xylanase in broiler barely-based diet the chymotrypsin and lipase activities in pancreas were increased and feed conversion was also improved (*P* <0.001) [28]. In our study, the addition of XG improved the activities of trypsin, chymotrypsin and amylase at 21d and 42d, while BM improved the activity of trypsin at 21d, but decreased at 42d, and AG improved the activity of chymotrypsin at 21d. The improvement of trypsin and chymotrypsin implied that the amount of protein available for digestion was increased by exogenous enzymes supplementation, and the digestibility of carbohydrate and amylum in the diet was also improved by XG, but lipase activity was not improved (except for AG in NC diet and XG in PC diet at 21d) in our study, which demonstrated that the addition BM showed few effect on the utilization of lipid in the diet, which was consistent with the result of AME and TME. Additionally, our study suggested that the exogenous enzymes showed more effects on the activities of endogenous digestible enzyme at 21d than that at 42d, which implied that the exogenous enzymes probably made up some deficiency of endogenous enzyme in the starter period. The present study demonstrated that XG decreased the feed cost per kg body weight gain, increased the profits in PC or NC diet. Furthermore, the addition of XG decreased the output of excrement, which plays an important role in the environmental protection.

In conclusion, supplementation of 0.02% BM or 0.01% AG or 0.05% XG could improve broiler diet feed conversion in corn-soybean meal diet by improving energy utilization and digestive physiology, and also supplementation of 0.05% XG had a preferable efficacy in low energy diet.

## Competing interests

The authors declared that they have no competing interests.

## Authors’ contributions

ZZ and Z conceived the study; ZZ, Z, D and B designed the study; Z raise the animals and carried out the lab analysis; Z, Z, Z, D and B contributed to data analysis; Z and Z wrote the manuscript. All authors read and approved the manuscript.
